# Dyskeratosis congenita

**DOI:** 10.4322/acr.2020.203

**Published:** 2020-09-02

**Authors:** Lorenzo Gitto, Robert Stoppacher, Timothy Eric Richardson, Serenella Serinelli

**Affiliations:** 1 State University of New York, Upstate Medical University, Department of Pathology, Syracuse, NY, USA

**Keywords:** Autopsy, Dyskeratosis Congenita, Hematopoiesis, Extramedullary, Pathology, Telomere Shortening

## Abstract

Dyskeratosis congenita (DC) is a genetic syndrome with progressive multisystem involvement classically characterized by the clinical triad of oral leukoplakia, nail dystrophy, and reticular hyperpigmentation. Frequent complications are bone marrow failure, increased rate of malignancy, lung and liver diseases. DC results from an anomalous progressive shortening of telomeres resulting in DNA replication problems inducing replicative senescence. We report a death due to DC in a 16-year-old male with bone marrow failure and multiple organ dysfunction. At autopsy, nail dystrophy and skin hypopigmentation were observed. Gross and microscopic examinations of the internal organs showed cardiac hypertrophy, multiple lung consolidations and prominent interstitial fibrosis, liver cirrhosis, and fibrosis. Multiple foci of extramedullary hematopoiesis were identified, including on the epidural surface of the dura, that is an infrequent location, mimicking a focal area of epidural hemorrhage. Only a few autopsy studies about DC are reported in the literature. Further research should be done to understand the pathophysiology of the disease and its complications.

## INTRODUCTION

Dyskeratosis congenita (DC) (also called Zinsser-Engman-Cole syndrome or short telomere disease) is a rare inherited genetic condition presenting with multisystem involvement. It was firstly reported by Zinsser in 1906,^1^ and then by Engmann in 1926,^2^ and Cole in 1930,^3^ giving the original eponym to the syndrome. The syndrome shows a typical triad of oral leukoplakia, nail dystrophy, and reticular hyperpigmentation,^4^ and it is frequently complicated by malignancy and bone marrow failure. Males are more affected than females since the inheritance is classically x-linked recessive. DC is characterized by the presence of short age-adjusted telomeres due to specific gene mutations, including *TINF2, TERC, TERT*, *C16orf57, NOLA2, NOLA3, WRAP53/TCAB1*, and *RTEL1*.^5^ The involved genes also encode for proteins that control critical steps of dividing cell maturation. Thus, their instability may also induce carcinogenesis.

Herein, we report a death due to DC in a 16-year-old boy with multiple organ dysfunction in which a full postmortem with neuropathology examinations were done. The significant clinical, radiological, and pathologic findings are presented, together with a review of the autopsy literature.

## CASE REPORT

**Figure 1:**
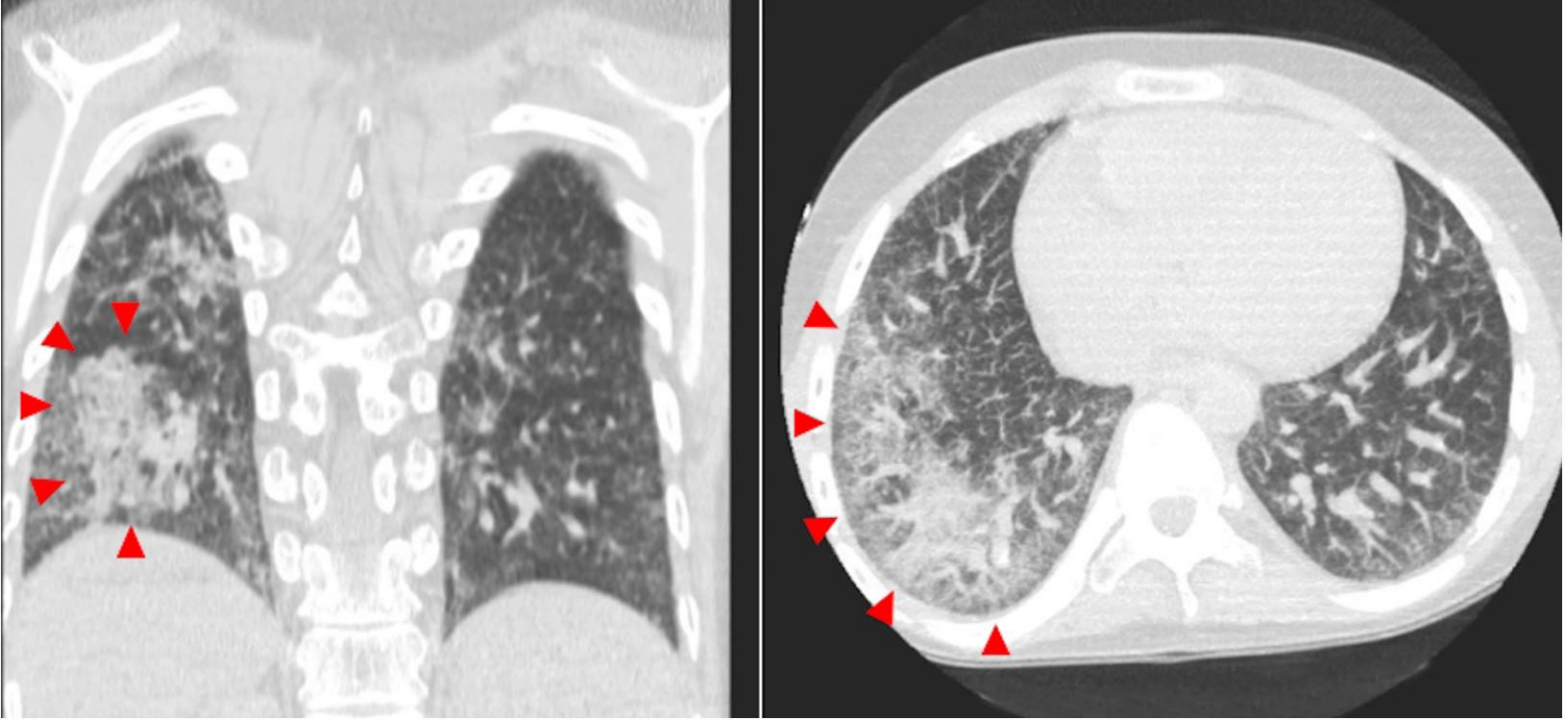
CT scan of the chest showing extensive reticulation, interlobular septal thickening, and patchy confluent areas of peripheral airspace consolidation on the right lung (red arrowheads).

A 16-year-old boy with a known medical history of DC due to *TINF2* mutation was admitted to the pediatric intensive care unit due to worsening respiratory distress and hypoxia. Since the diagnosis, his clinical course was complicated by bone marrow failure status post-transplant with persistent pancytopenia, nodular regenerative hyperplasia of the liver, hypersplenism with portal hypertension, chronic gastrointestinal bleeding due to small vascular malformations of the intestine requiring constant blood transfusions, and restrictive lung disease. The recent onset of respiratory distress was related to the development of hepatopulmonary syndrome. The physical examination disclosed a thin, ill-appearing, tachypneic (44/min), and tachycardic (153 bpm) adolescent in moderate respiratory distress with a high fever (38.5 C) and decreased breath sound in the right lower lung fields. Generalized dry and mottled appearing skin with prominent veins and pallor was present. No abdominal distension or tenderness, or edema were observed. A CT scan of the chest showed extensive reticulation, interlobular septal thickening, signs of pulmonary hypertension, and patchy confluent areas of perihilar and peripheral airspace disease, mainly on the right lung (Figure 1).

Blood analyses showed pancytopenia and abnormal liver function tests. Oxygen administration was started, and blood and sputum cultures were drawn. Empiric antibiotic therapy was then administered. Despite adequate treatment, the patient’s respiratory conditions progressively worsened, and he eventually died two days later. Cultures obtained for bacteria, fungi and viruses were negative. An autopsy was requested.

## AUTOPSY FINDINGS

**Figure 2:**
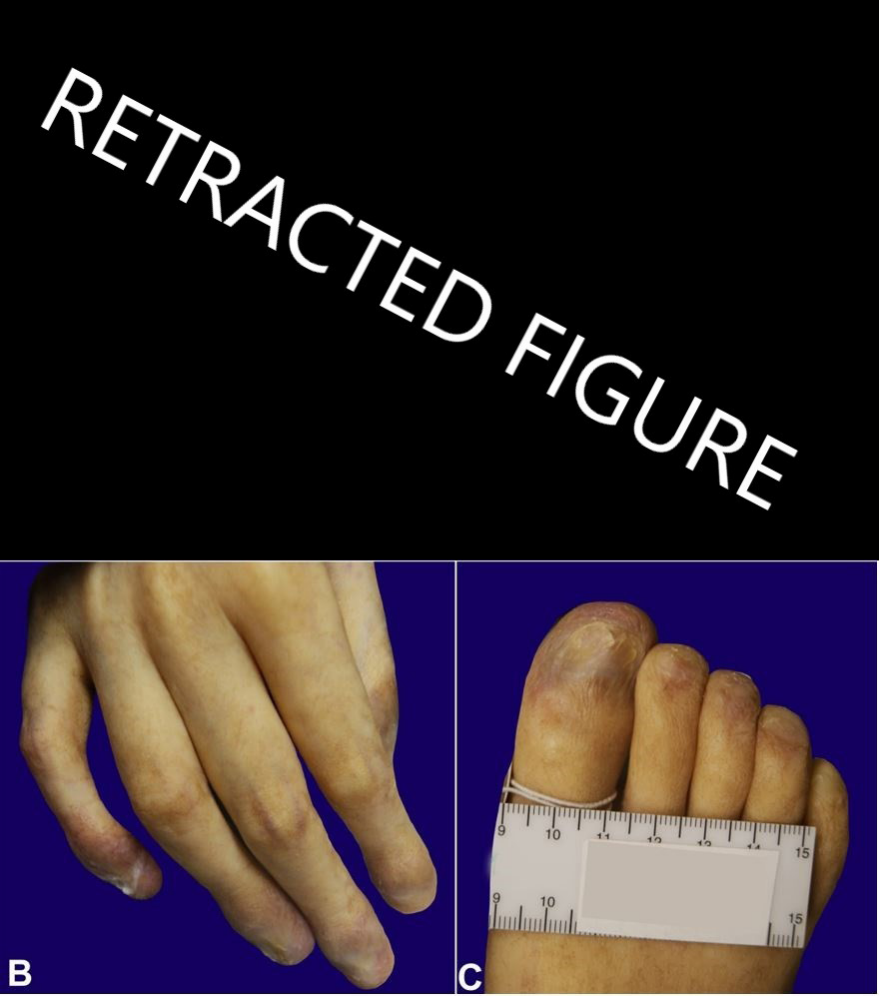
**A –** Macular areas of hypopigmentation with a reticulated pattern observed at the external examination (fig. 2). **B and C –** Note the absence of nails with exposed nailbed and dystrophic nails.

At autopsy, the body was of a Caucasian male, weighing 44 kg and measuring 162 cm. External examination demonstrated tan macular areas of hypopigmentation with a reticulated pattern over the neck, torso, and extremities (Figure 2A). The finger and toenails were absent, exposing the nailbeds, except for the 1st and 4th right toenails, which were dystrophic and thickened (Figure 22C).

**Figure 3:**
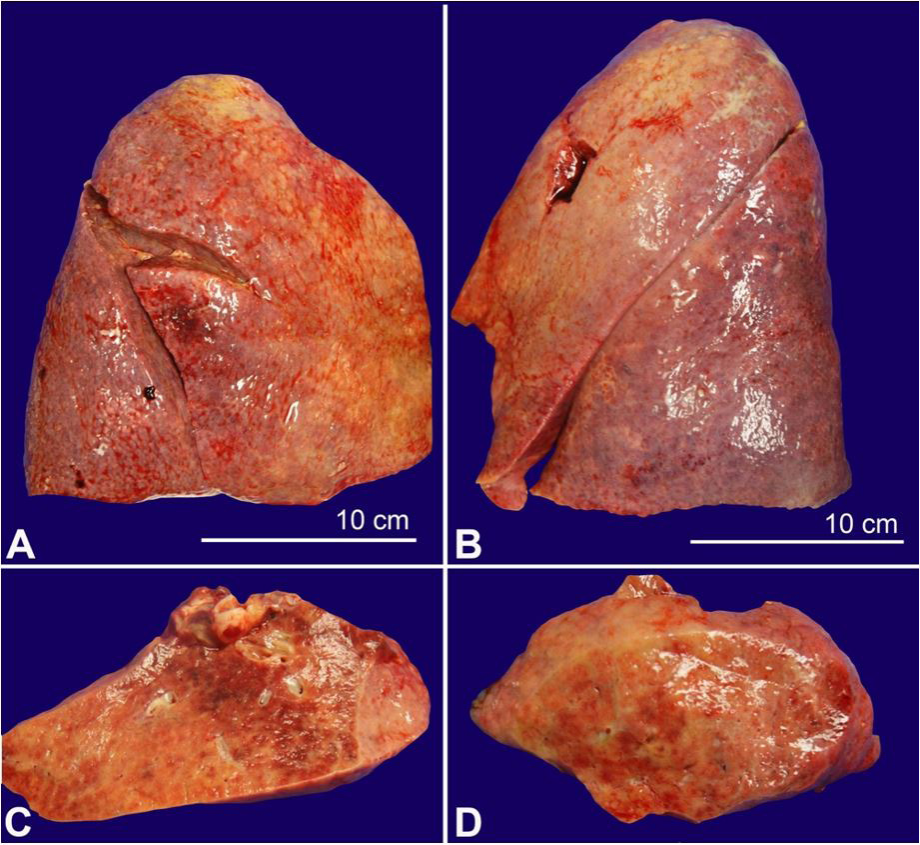
Lungs with firm overall parenchyma with multiple areas of consolidation. **A –** Right lung. **B –** Left lung. **C and D –** Left and right lung sections.

**Figure 4:**
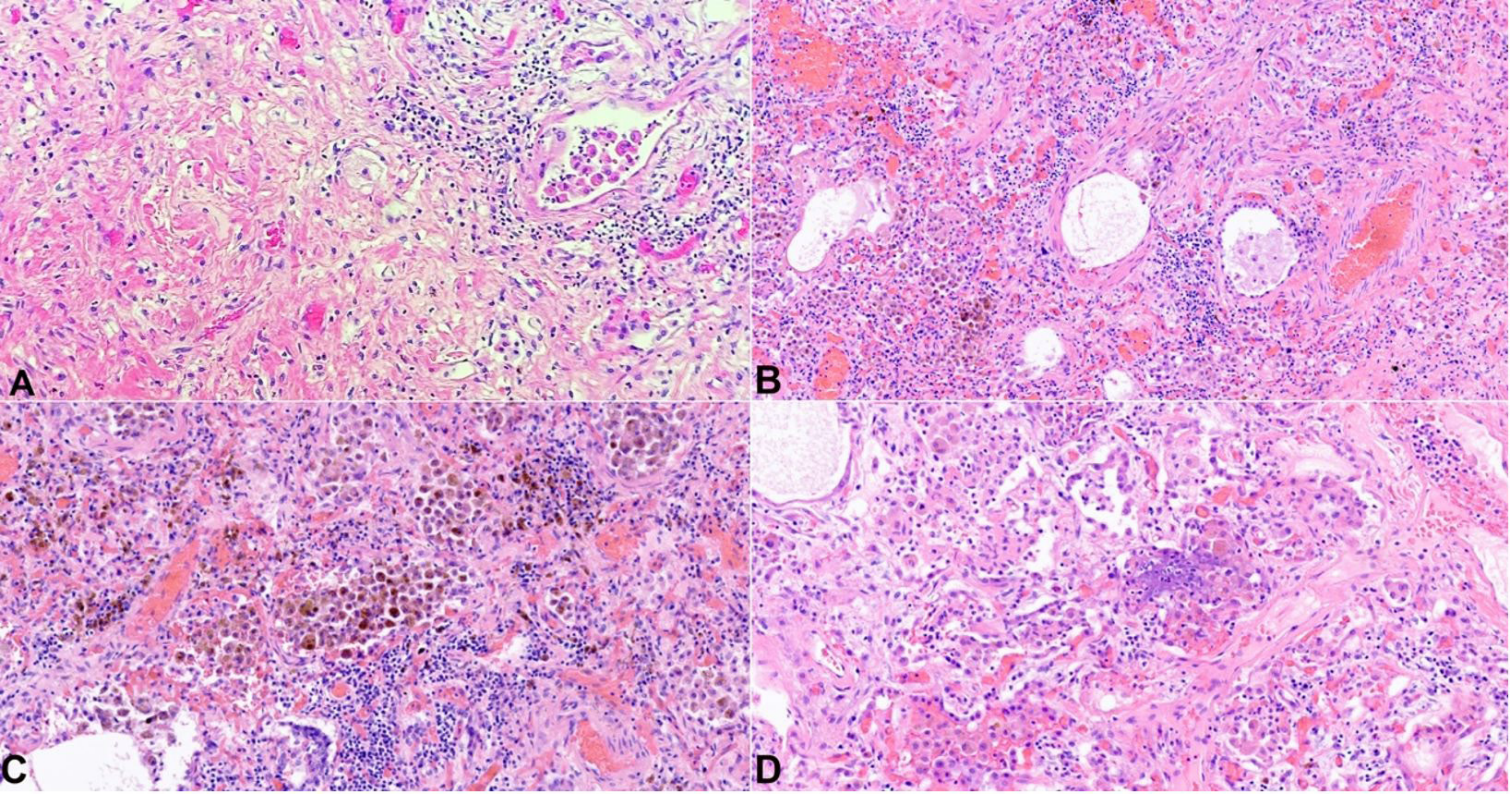
Photomicrographs of the lungs. **A –** Prominent fibrosis with increased dilated vascular channels (H&E, 100x); **B –** Mild thickening of the pulmonary arterioles, consistent with pulmonary hypertension (H&E, 100x); **C and D –** Diffuse alveolar damage with acute and organizing pneumonia in addition to numerous hemosiderin-laden macrophages are present (both H&E, 200x).

On internal examination, both lungs showed multiple areas of consolidation, mainly in the right lung (Figure 3). Postmortem lung cultures were collected in the attempt to detect potential etiologic agents, but no viral or bacterial organisms were isolated. Histology of lung samples showed diffuse alveolar damage with acute and organizing pneumonia in addition to thickened pulmonary arteries consistent with pulmonary hypertension. The lower lobe of the right lung showed more prominent fibrosis with increased dilated vascular channels. (Figure 4).

**Figure 5:**
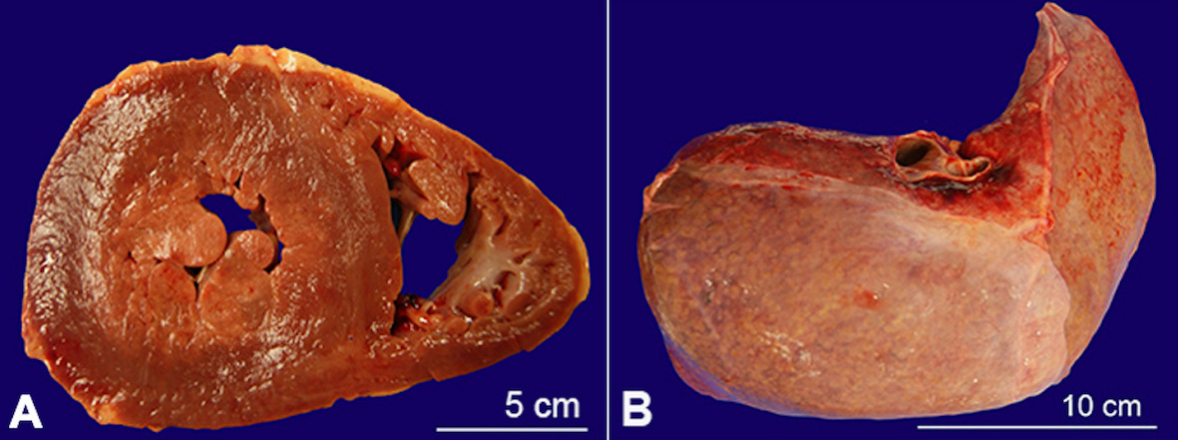
Gross view of the: **A –** Left and right ventricular hypertrophy; **B –** liver with firm granular parenchyma, with gross areas of nodule formation.

Cardiac examination revealed both left and right ventricular hypertrophy. The hearth weight was 400 g (mean reference range [mRR]: 183 gr), and the myocardium showed the following thicknesses: left ventricle - 1.7 cm (mRR: 0.79 cm); interventricular septum - 1.6 cm (mRR:0.77 cm); right ventricle hypertrophy - 0.5 cm. The right ventricle hypertrophy was consistent with long-standing pulmonary hypertension (Figure 5A).

The liver parenchyma was firm and granular, with gross areas of nodule formation (Figure 5B). Scattered dilated vascular channels throughout the parenchyma were present.

**Figure 6:**
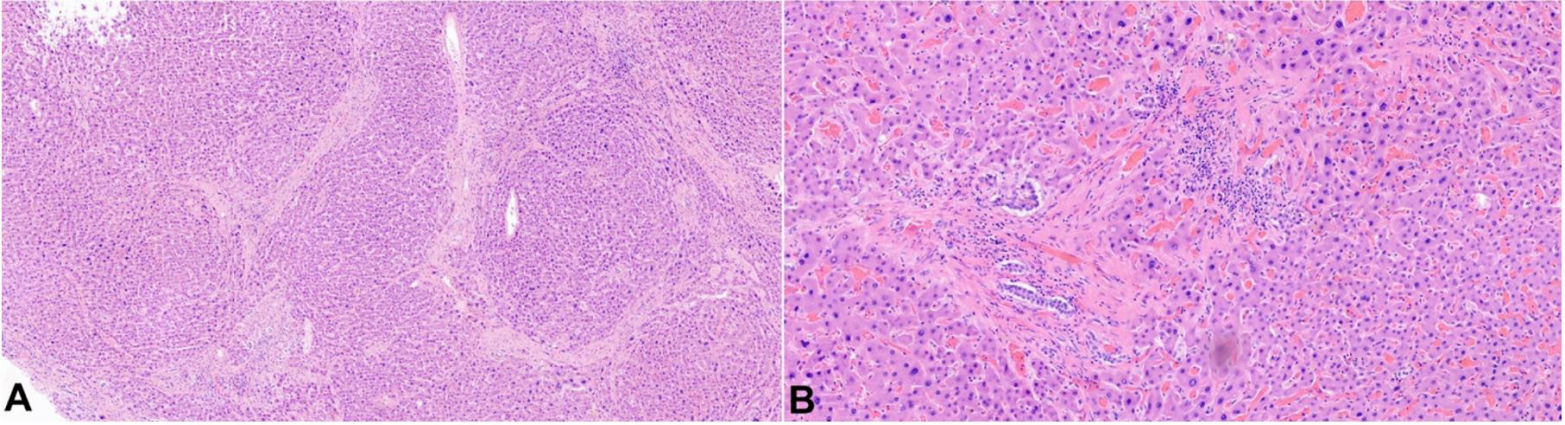
Photomicrographs of the liver with: **A –** focal areas of bridging fibrosis and nodule formation (H&E, 40X); **B –** Biliary duct proliferation and patchy increased chronic inflammation observed in other portal triads (H&E, 40X).

Microscopic examination of the liver (Figure 6) showed variable expansion of the portal triads by fibrosis, with focal areas of bridging fibrosis and nodule formation. In other portal triads, there was biliary duct proliferation and patchy increased chronic inflammation.

**Figure 7:**
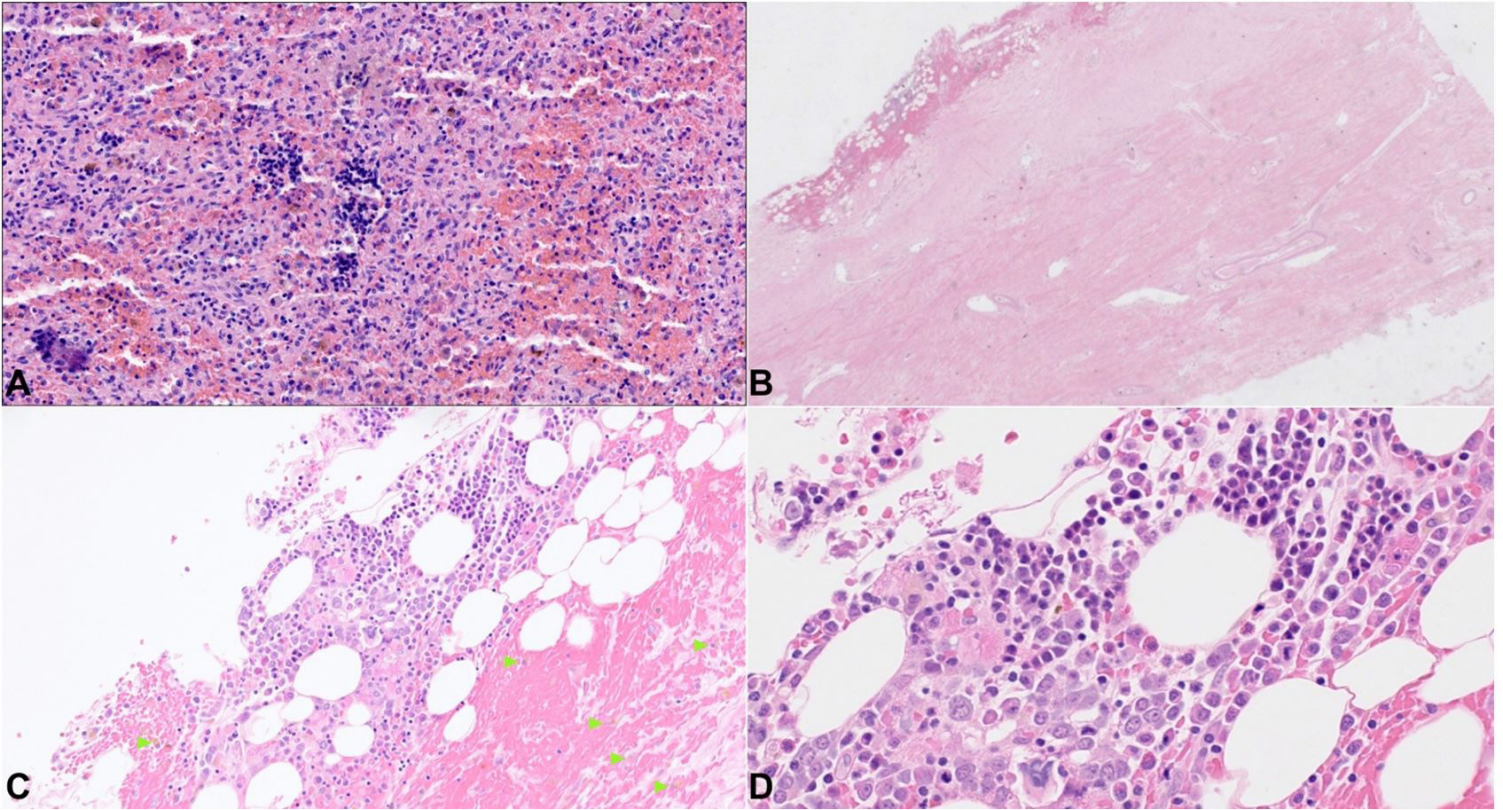
Photomicrographs of the: **A –** Spleen with foci of extramedullary hematopoiesis and lipid-laden macrophages; **B –** dura mater with small area of epidural hemorrhage (H&E, 20X) and in **C –** scattered hemosiderin-laden macrophages(H&E, 100X; green arrowheads); **D –** within the hemorrhagic area, numerous erythroid precursors, megakaryocytes, and other immature hematolymphoid cells are present, consistent with trilineage extramedullary hematopoiesis (H&E, 400X).

The spleen was increased in size and weighed 550 g (RR: 112 gr). Microscopic examination of the parenchyma showed an expansion of the red pulp and a relative decrease in the white pulp. Foci of extramedullary hematopoiesis and lipid-laden macrophages were observed (Figure 7A

).

Examination of the brain showed very mild cerebellar atrophy and focal red-brown epidural discoloration at the midline of the epidural surface, suspicious for epidural hemorrhage. Histologic examination of dura mater sections showed small areas of adherent hemorrhage and scattered hemosiderin-laden macrophages. Within the hemorrhagic area, there was a collection of erythroid precursors, megakaryocytes, and other immature hematolymphoid cells, consistent with trilineage extramedullary hematopoiesis (Figure 7BD).

The death was due to DC complicated by multiple organ involvement and terminal respiratory failure.

## DISCUSSION

Telomeres are fragments of DNA present at the ends of chromosomes, consisting of tandem repeats of the sequence 5′-TTAGGG-3′. Telomeres confer genomic stability by protecting the chromosomes during DNA replication and prevent the ends of chromosomes from sticking to each other. The progressive shortening of telomeres induces the replicative senescence, preventing further the cell division. The average telomere length is 11 kilobases in the newborn and decreases to 4 kilobases in the elderly.^6^ ^,^ ^7^ Their functions are regulated by the telomerase complex, which is responsible for adding telomeric repeats to the ends of chromosomes. While telomeres shortening is a physiologic phenomenon associated with aging, there are genetic conditions that can cause premature shortening, leading to diseases. A defect in telomere maintenance or telomerase activity may result in DNA replication problems, leading to carcinogenesis or chronic, severe disease, such as dyskeratosis congenita.^8^

Numerous genes have been shown to be mutated in DC. In the presented case, the patient was affected by *TINF2* gene mutation. The *TINF2* gene encodes for TIN2, a subunit of the six-protein shelterin/telosome complex involved in the regulation of telomere length and protection. TIN2 plays a central role in the assembly and function of the complex, connecting three primary DNA-binding proteins, TRF1, TRF2, and TPP1/POT1, thus stabilizing telomeres.^9^ ^,^ ^10^

The resulting clinical phenotype can be variable. Nail changes are characterized by thin, dystrophic nails that may be markedly shortened and fragile with a subungual thickening. The dystrophy usually appears in the first decade of life and begins as longitudinal ridging and splitting. With the progression of the disease, it eventually leads to pterygium formation with the distal expansion of the hyponychium and obliteration of the distal groove. This leads to a progressive loss of the distal part of the nail, and almost total nail loss can occur.^11^

Skin findings can be variable, ranging from tan-to-gray macular or patchy areas of hyper or hypopigmentation with a reticulated or mottled pattern. Atrophy and telangiectasias can also be present (poikiloderma). A typical distribution over the sun-exposed areas, including the upper trunk and the extremities, is observed.^12^ DC is also frequently associated with oral findings, which include oral leukoplakia, increased dental caries, hypodontia, thin enamel structure, aggressive periodontitis, intraoral brown pigmentation, tooth loss, taurodontism, and blunted roots.^13^ Another frequent finding is bone marrow failure. It usually develops before the age of 20 years, resulting in chronic pancytopenia. Presumably, chronic bone marrow failure is due to the premature shortening of telomeres, which reduces the proliferation capacities of the hematopoietic stem cells.^14^

In the current case, there was nail dystrophy and widespread hypopigmentation of the skin involving the neck, torso, and extremities, but no definitive oral lesions were detected. Although the clinical triad is classically present, there can be cases in which the subject lacks one of the clinical features, including oral findings.^15^

A remote amputation of the left 4th distal phalanx was also observed. The subject previously developed a hyperkeratotic mass on the tip of the fourth finger of the left hand. The mass was excised, and microscopic examination showed morphological features consistent with squamous cell carcinoma in situ with verrucous architecture. It is known that due to telomeres dysfunction, carcinogenesis can occur, and various types of malignancies can develop in patients with DC.^16^

Even if DC is rarely seen in the forensic setting, the severe and progressive skin alterations typical for this syndrome may lead to adermatoglyphia, which is the total or partial loss of fingerprints. The absence or modification of the fingerprints may have important medicolegal implications, especially for identification purposes.

The subject also suffered from a chronic bone marrow failure, requiring a bone marrow transplant. Despite that, chronic, severe, and persistent pancytopenia developed, requiring a frequent transfusion regimen. In this case, an interesting microscopic finding was the presence of multiple foci of extramedullary hematopoiesis (EMH) in different organs. While some of them were found in the spleen, which is a common location for EMH, multiple foci were detected on the epidural surface, which is an exceptionally rare location for EMH, mimicking a focal epidural hemorrhage.

EMH is defined as hematopoietic elements identified outside the bone marrow. It is considered a compensatory process due to chronic bone marrow suppression leading to hematopoietic deficiency. While subdural EMH is relatively common, epidural EMH is rare, and the falx cerebri is the most commonly involved dural site. The pathogenesis of dural EMH is unknown. Several theories have been postulated, including the persistence of primitive dural rests with hematopoietic capacity, embolization of hematopoietic cells, or direct extension of bone marrow from bone fractures into the epidural space.^17^

Epidural hemorrhages (EDHs) are a common posttraumatic finding, generally occurring as a consequence of skull fractures with a secondary middle meningeal arterial laceration. Spontaneous intracranial EDHs have been described in patients with intracranial infections, coagulation disorders, vascular malformation, and neoplasms. In the presented case, there was no history of head trauma or intracranial pathology. The EMH was likely due to the chronic bone marrow failure with persistent pancytopenia, which is one of the primary manifestations of dyskeratosis congenita. The resulting EDH was an incidental finding.

Another relevant abnormality that was observed in our case was the extensive pulmonary fibrosis. The lung showed multiple areas of consolidation, and prominent fibrosis with increased dilated vascular channels was found at the histology, likely representing healed areas of prior insults. The lung is commonly involved in a patient with DC, with lung fibrosis being one of the most common pathologic findings. Lung fibrosis has also been associated with mutations of the *TINF2* gene. The mechanism that leads to lung fibrosis is still not completely understood. One theory is that the potential shortening of telomeres in the alveolar epithelial cells can lead to an aberrant lung repair process by their premature and enhanced apoptosis, causing pulmonary fibrosis.^18^ ^,^ ^19^

Finally, a small percentage of patients with DC also develop hepatic involvement, with hepatocellular necrosis and liver cirrhosis being the most common entities. A possible cause for this finding is the chronic transfusion regimen due to bone marrow failure, which can lead to iron overload and hemochromatosis. Progressive liver disease can eventually lead to hepatopulmonary syndrome, which is an important cause of hypoxemia and is characterized by a high mortality rate.^20^ ^,^ ^21^

A review of the autopsy literature was done via PubMed and Google Scholar search, using the following keywords: “Dyskeratosis Congenita”, “Zinsser-Engman-Cole syndrome”, “Autopsy”, and “postmortem examination”. Only papers in the English language in which an autopsy was performed were considered. A total of 7 articles was retrieved in the online databases.^22^ ^-^ ^28^ In the reported cases, subjects were all males, and their ages ranged from 6 to 39 years. In all cases, dermatological findings were a consistent classic triad of DC. Internal findings were slightly different from case to case, but spleen, lung, and liver involvement were commonly observed. A summary of the literature review, including the significant autopsy findings, is reported in Table 1.

**Table 1:** Reported autopsy cases of dyskeratosis congenita in the English literature.

**Author (year)**	**Sex**	**Age**	**Autopsy findings**
Bryan et al.^22^	M	31	**EE**: Moderately clubbed fingers and toes, with softening of the nailbeds. **IE**: Portal cirrhosis, esophageal and mediastinal varices, chronic pneumonitis, patent foramen ovale, mild hypoplasia of the aorta, and anomalous ostia of the left coronary artery.
Trowbridge et al.^23^	M	29	EE: “features consistent with dyskeratosis congenita”. **IE**: Markedly hypocellular bone marrow. Sections of mediastinal, periaortic, and mesenteric lymph nodes showed striking lymphoid depletion of cortical and pericortical areas. Primary follicles were almost totally absent from the cortex. Normal liver and spleen. Disseminated cytomegalovirus infection.
Mills et al.^24^	M	14	**EE**: Dystrophic and fragmented nails, patchy leukoplakia on the buccal mucosa, marked hyperkeratosis and hyperhidrosis of the palms and soles, atrophic skin of the hands and feet. **IE**: Acute cardiac failure and gastroenteritis. Extensive osteoporosis and marked erythroid hyperplasia. Enlarged parathyroid glands. Brain and cerebellar calcifications. Microscopic sections of brain showed laminated, amorphous, perivascular concretions that condensed to form large irregular masses.
Wiedemann et al.^25^	M	22	**EE**: Absent fingernails and toenails, fine reticular hyperpigmentation over the face and neck. **IE**: No thymic tissue identified. Histologically, the spleen and lymph nodes contained numerous plasma cells, but there was pericortical lymphoid depletion of pericortical areas. Massive disseminated candidiasis with involvement of kidney, liver, bone marrow, lung, trachea, heart, and skeletal muscle.
Kawaguchi et al.^26^	M	24	**EE**: Reticular hyperpigmentation of the skin studded with depigmented spots, dystrophy of the nails, atrophy of lingual papillae, and hypertrophic squamous epithelium with cellular atypia in the oral and anal mucosa. **IE**: Scirrhous poorly differentiated adenocarcinoma with irregular ulceration of the lower part of the rectum, involving its entire wall and the surrounding tissues, including the urinary bladder and the prostate. Fibrocongestive splenomegaly (450 gr.). Mild esophageal varices. Markedly atrophic testes. Normocellular bone marrow with erythroid hyperplasia. Extremely slender folia of the cerebellum. Atrophy of the frontal lobes of the brain. Marked lymphocyte depletion and atrophy of lymphoid parenchyma in systemic lymph nodes, tonsils, gastrointestinal tract, spleen, and thymus.
Verra et al.^27^	M	39	**EE**: Hyperpigmentation, forming a network pattern, over the neck, the chest and around the armpits. Strongly dystrophic fingernails and toenails, either destroyed or fused with keratotic skin. Tongue leukoplasia. **IE**: (autopsy restricted to the right lung): Lung nonspecific collagenous interstitial fibrosis with cuboidal metaplasia of alveolar epithelial cells in an area devoid of bronchiectasis.
Rocha et al.^28^	M	6	**EE**: Dystrophic nails, leucoplakia, skin pigmentation, epiphora. **IE**: Liver veno-occlusive disease. Lung concentric subintimal thickening and lumen narrowing by fibrosis of terminal lung venules. Kidneys thrombotic micro-angiopathic arterial syndrome.

EE: external examination; IE: internal examination.

DC is a rare genetic syndrome with multisystem involvement. The leading causes of death in patients with DC are bone marrow failure, respiratory distress due to severe pulmonary disease, malignancies, and less commonly hepatopulmonary syndrome. Many DC pathophysiologic mechanisms are still not completely understood. Since autopsy studies in cases of DC are rarely reported in the literature, further research in the pathology field should be done to clarify the pathophysiology of the disease and its complications.
