# Partial retraction of “Dyskeratosis congenita” [Autops Case Rep 10(3) (2020) e2020203]

**DOI:** 10.4322/acr.2021.341

**Published:** 2021-11-01

Gitto L, Stoppacher R, Richardson TE, Serinelli S. Dyskeratosis congenita. Autops Case Rep [Internet]. 2020 Jul-Sep;10(3):e2020203. https://doi.org/10.4322/acr.2020.203

The reason for retracting this image is that, although the authors obtained authorization from the deceased’s next-of-kin to perform the autopsy and use the data for publication, this specific picture should not have been included.

This issue was brought to the attention of the journal's Editor-in-chief by the paper's corresponding author. After exchanging correspondence with the authors and their institution, the Editor-in-chief obtained a formal letter from the Pathology department at SUNY Upstate Medical University requesting the retraction. The letter states that the four authors of the paper are in agreement with this decision and also that this issue was brought to the attention of the Ethics Committee at the SUNY Upstate Medical University who supports the image retraction.

The Editorial Board and the authors would like to express their most sincere apologies to the family members for this unintentional oversight by the authors.

Prof. Dr. Fernando Peixoto Ferraz de Campos

Editor-in-chief

