# ERRATUM: Partial retraction of “Dyskeratosis congenita” [Autops Case Rep 10(3) (2020) e2020203]

**DOI:** 10.4322/acr.2021.349

**Published:** 2022-02-11

Due to a desktop publishing error, the partial retraction notice “Partial retraction of “Dyskeratosis congenita” [Autops Case Rep 10(3) (2020) e2020203]” (DOI https://doi.org/10.4322/acr.2021.341), published in Autops. Case Rep. 11, 2021, was published with an error.

Where the text reads:

Gitto L, Stoppacher R, Richardson TE, Serinelli S. Dyskeratosis congenita. Autops Case Rep [Internet]. 2021;11:e2021341. https://doi.org/10.4322/acr.2021.341

It should read:

Gitto L, Stoppacher R, Richardson TE, Serinelli S. Dyskeratosis congenita. Autops Case Rep [Internet]. 2020 Jul-Sep;10(3):e2020203. https://doi.org/10.4322/acr.2020.203

The publisher apologizes for the errors.

